# Seasonal variation in muscle sympathetic nerve activity

**DOI:** 10.14814/phy2.12492

**Published:** 2015-08-11

**Authors:** Jian Cui, Matthew D Muller, Cheryl Blaha, Allen R Kunselman, Lawrence I Sinoway

**Affiliations:** 1 Penn State Hershey Heart and Vascular Institute, Pennsylvania State University College of Medicine, Milton S. Hershey Medical CenterHershey, Pennsylvania; 2 Division of Biostatistics and Bioinformatics, Department of Public Health Sciences, Pennsylvania State University College of Medicine, Milton S. Hershey Medical CenterHershey, Pennsylvania

**Keywords:** Cardiovascular diseases, hemodynamic, nervous system, risk factors, sympathetic

## Abstract

Epidemiologic data suggest there are seasonal variations in the incidence of severe cardiac events with peak levels being evident in the winter. Whether autonomic indices including muscle sympathetic nerve activity (MSNA) vary with season remains unclear. In this report, we tested the hypothesis that resting MSNA varies with the seasons of the year with peak levels evident in the winter. We analyzed the supine resting MSNA in 60 healthy subjects. Each subject was studied during two, three, or four seasons (total 237 visits). MSNA burst rate in the winter (21.0 ± 6.8 burst/min, mean ± SD) was significantly greater than in the summer (13.5 ± 5.8 burst/min, *P < *0.001), the spring (17.1 ± 9.0 burst/min, *P = *0.03), and the fall (17.9 ± 7.7 burst/min, *P = *0.002). There was no significant difference in MSNA for other seasonal comparisons. The results suggest that resting sympathetic nerve activity varies along the seasons, with peak levels evident in the winter. We speculate that the seasonal changes in sympathetic activity may be a contribution to the previously observed seasonal variations in cardiovascular morbidity and mortality.

## Introduction

Large epidemiological studies from many different countries suggest greater cardiovascular mortality and hospitalization rates in winter than in other seasons (Dunnigan et al. 1970; Boulay et al. 1999; Kloner et al. 1999; Sheth et al. 1999; Stewart et al. 2002). For example, the mortality and the incidence of heart failure are higher in the winter than during other seasons (Boulay et al. 1999; Martinez-Selles et al. 2002; Stewart et al. 2002). This effect of the winter is seen in both the northern hemisphere (Boulay et al. 1999; Martinez-Selles et al. 2002; Stewart et al. 2002) and southern hemisphere (Diaz et al. 2007). The causes of this seasonal effect are not known but may be due to variations in cardiovascular disease risk factors (Hopstock et al. 2013). For example, epidemiological data have shown that blood pressure (BP) is higher in the winter than in the summer (Alperovitch et al. 2009; Modesti et al. 2013), a seasonal effect that may increase with age (Brennan et al. 1982; Woodhouse et al. 1993; Alperovitch et al. 2009). It has been suggested by some that this effect may be mediated by differences in the environmental temperature (Woodhouse et al. 1993; Alperovitch et al. 2009; Kent et al. 2011; Modesti et al. 2013). However, the pathophysiologic processes that mediate the seasonal differences in BP are not known.

It is thought that increase in sympathetic activity plays a critical role in the pathogenesis of hypertension as well as congestive heart failure. Specifically, it is known that hypertensive individuals can have high levels of resting muscle sympathetic nerve activity (MSNA) and plasma norepinephrine (Grassi et al. 2003). Moreover, it is clear that MSNA and catechol levels are higher in heart failure subjects than in controls (Grassi et al. 2003). It should be noted that high concentration of catecholamines can worsen cardiac performance, lead to atrial and ventricular arrhythmias, and evoke muscles, splanchnic, and renal vasoconstriction (Packer 1988). Thus, it is not surprising that elevated levels of norepinephrine and/or basal MSNA are associated with increased cardiac mortality (Cohn et al. 1984; Barretto et al. 2009).

If seasonal variations in cardiovascular morbidity and mortality are linked with seasonal differences in sympathetic tone, than we would expect that basal sympathetic nerve activity in normal controls should vary as a function of season. A prior short report with eight subjects (Niimi et al. 1999) suggested that MSNA in the winter might be greater than the summer. In the present study, we hypothesized that MSNA would vary with the seasons of the year with peak levels evident in the winter.

## Methods

### Subjects

In this report, we retrospectively analyzed the baseline data of 237 visits from a total of 60 healthy adult volunteers in the past reports (Cui et al. 2006, 2007, 2008a, 2008b, 2009, 2010, 2011a, 2011b, 2012a, 2012b, 2013) as well as from unpublished pilot studies. The 60 healthy adult volunteers (35 male, 25 female) were from central Pennsylvania. The subjects involved in this report were indoor workers or medical students. Thus, it is unlikely that their occupational activity varies as a function of seasons. There were no collegiate, semiprofessional or professional athletes in the 60 subjects. None of data presented were obtained during exercise training studies. The subjects were studied during two (*N *=* *37), three (*N *=* *16), or four (*N *=* *7) seasons in our laboratory from June 2005 to May 2013 (total 237 studies). The mean time difference between visits within the individuals was 0.5 ± 0.6 years. The maximal time difference between visits within the individuals was 2 years.

All subjects were of normal height (175 ± 9 cm, mean ± SD) and weight. The ages of the subjects (21.5–63.5 years) in each season in Table1 were accurate to the month of the study. All subjects were normotensive, in good health, and none were taking medications. Subjects refrained from caffeine, alcohol, and exercise for 24 h prior to the study. All subjects had a light breakfast or a light lunch before the study. All experimental protocols used to obtain these resting data were approved by the Institutional Review Board of the Milton S. Hershey Medical Center and conformed with the Declaration of Helsinki. Written informed consent was obtained from each subject.

**Table 1:** Age and weight in the four seasons

	Spring	Summer	Fall	Winter	*P*
Age (years)					
All	29.1 ± 9.8	28.5 ± 9.2	29.3 ± 10.7	28.4 ± 8.7	0.660
F	26.6 ± 3.1	26.3 ± 1.8	25.6 ± 2.6	26.6 ± 3.1	0.768
M	31.4 ± 12.9	31.1 ± 12.0	31.5 ± 12.9	29.6 ± 10.8	0.617
Weight (kg)					
All	72.2 ± 13.3	75.2 ± 13.0	77.2 ± 13.3	76.2 ± 12.5	0.225
F	65.2 ± 9.2	68.8 ± 10.5	65.9 ± 11.8	66.2 ± 9.2	0.178
M	79.9 ± 13.3	79.9 ± 12.7	83.9 ± 9.0	82.9 ± 9.8	0.942
BMI (kg/m^2^)					
All	24.2 ± 3.2	24.7 ± 3.0	25.0 ± 2.9	24.6 ± 2.6	0.317
F	23.4 ± 2.8	23.9 ± 2.5	23.4 ± 2.6	23.1 ± 2.1	0.249
M	25.1 ± 3.5	25.3 ± 3.3	25.9 ± 2.7	25.5 ± 2.5	0.895
F/M	15/15	17/23	14/24	17/26	
N					
All	30	40	38	43	

Data are reported as means ± SD.

*P: P* value for the seasons.

### Measurements

A standard three-lead electrocardiogram (Cardicap*/*5, Datex-Ohmeda, GE Healthcare, NJ) was recorded. Systolic (SBP), diastolic (DBP), and mean arterial pressure (MAP) was obtained from the brachial artery with an automated sphygmomanometer (Dinamap, Critikon, Tampa, FL, or Philips SureSigns Vs3, Andover, MA). Other noninvasive measurements (e.g., skin blood flow with Laser Doppler) differed in the different projects. Those data were not included in this report. In most (206/237) studies, subjects received an intravenous catheter into a forearm vein at the beginning of the instrumentation. Other invasive measurements such as intra-arterial catheter were not employed in any of the included studies.

As described in our previous reports (Cui et al. 2006), multifiber recordings of MSNA were obtained with a tungsten microelectrode inserted in the peroneal nerve. A reference electrode was placed subcutaneously 2–3 cm from the recording electrode. The recording electrode was adjusted until a site was found in which muscle sympathetic bursts were clearly identified using previously established criteria (Vallbo et al. 1979). The nerve signal was amplified, a band-pass filtered with a bandwidth of 500–5000 Hz, and integrated with a time constant of 0.1 sec (Iowa Bioengineering, Iowa City, IA). The nerve signal was also routed to a loudspeaker and a computer for monitoring. All these microneurographic recordings were performed by one investigator using the same recording system with the same settings (gain, filter, etc.), and all data were obtained from the same laboratory.

### Protocol

All subjects were tested in the supine position in a temperature-controlled room (23 ± 1°C). After instrumentation, all subjects had a ∼5-min acclimation period before commencing data were collected. Subsequently, all variables were continuously recorded for 6 min. Cuff BP was obtained once or twice during this period. The 6-min baseline was at least 30 sec prior to any subsequent interventions (e.g., handgrip, etc.). It should be noted that the data collection started approximately 70–110 min after the subjects arrived in the laboratory. The data were collected during ∼9:00–10:30 am (209 visits) or during ∼1:30–3:00 pm (28 visits).

Outdoor temperatures from 1 June 2005 to 31 May 2013 were obtained from the records (http://www.wunderground.com/history/) of Middletown, Pennsylvania (∼13 km from the testing site). Based on the temperature records, in this project we considered April and May as spring (averaged daily high, mean, and low temperatures: 20.3 ± 3.2, 14.9 ± 3.0, and 9.1 ± 3.3°C), June, July, and August as summer (29.1 ± 1.5, 24.0 ± 1.5, and 18.8 ± 1.5°C), September, October, and November as fall (18.4 ± 5.7, 13.6 ± 5.5, and 8.4 ± 5.3°C), and December, January, February, and March as winter (6.5 ± 3.8, 2.2 ± 3.3, and −2.4 ± 2.8°C). These temperatures are the averaged numbers from the records from June 2005 to May 2013.

### Data analysis

Data were sampled at 200 Hz via a data acquisition system (MacLab, AD Instruments, Castle Hill, Australia). As described in our previous studies (Cui et al. 2006), MSNA bursts were first identified in real time by visual inspection of the data, coupled with the burst sound from the audio amplifier. These bursts were further evaluated by a computer program that identified bursts based on fixed criteria, including an appropriate latency following the R-wave of the electrocardiogram, and having a signal-to-noise ratio of at least 2:1. Although MSNA can be quantified as burst rate (i.e., bursts/min) and total activity (i.e., total burst area/min), only MSNA burst rate and burst incidence were analyzed in this report (Wallin 2007) for the following reasons. The burst rate (as well as the burst incidence) is the basic index for MSNA. When the MSNA bursts can be identified using standard criteria (Vallbo et al. 1979), recording quality has little influence on the measured bursts rate. In fact, a number of prior reports have shown that the MSNA burst rate is reproducible during repeated visits in healthy individuals (Fagius and Wallin 1993; Wallin 2007) and in diabetic patients (Hoffman et al. 1998). On the other hand, to further quantify MSNA changes in response to stimulation (e.g., cold pressor test), total activity (burst amplitude or burst area) is often calculated. Within a recording, this index can more precisely reflect the changes in sympathetic nerve activity than that observed using burst rate. However, the MSNA amplitude measurements (i.e., in volt or millivolt) are greatly affected by the relative position of the electrode tip in the sympathetic nerve bundle, even when recording device settings (gain, filter, etc.) are the same. Thus, “normalization” is often used when reporting MSNA total activity. In many prior reports, the baseline values of MSNA total activity were normalized to a relative value of 100 units/min. In other reports (Halliwill 2000; Cui et al. 2006), the normalization was accomplished by assigning the largest MSNA burst amplitude under resting conditions to 100 (or 1000). However, the normalized MSNA total activity can still be influenced by the recording quality. Therefore, considering all data in the present study were the baseline data collected from different visits, the MSNA total activity was not reported (Fagius and Wallin 1993; Wallin 2007).

Mean heart rate was obtained from the 6-min ECG. When the BP was measured more than once, the values were averaged. It should be noted that these basic data analyses were performed during many prior projects (Cui et al. 2006, 2007, 2008a, 2008b, 2009, 2010, 2011a, 2011b, 2012a, 2012b, 2013) by multiple investigators. These projects were not originally designed to evaluate the seasonal differences. The MSNA data for any season in the present report were collected during the performance of many projects. Moreover, all these projects were performed during several seasons or years, and thus the data from each individual project were distributed into several seasons in the present report. Thus, the other factors (e.g., instrumentation time, etc.) during the data collection and the data analysis were unlikely to have influences on the results in the present report.

If a subject was studied multiple times during a season (in the same or different years), all variables including age and weight were averaged within a season per subject. Thus, at most a single subject would provide four averaged assessments, that is, one per season, of a variable for analytical purposes.

### Statistics

A linear-mixed effects model (Verbeke and Molenberghs 2000), which is an extension of the analysis of variance model accounting for repeated measurements (i.e., season) per subject, was used to assess the association of season with the cardiovascular outcomes. Within the framework of the linear mixed-effects model, the Tukey–Kramer procedure was used to account for multiple comparisons testing between the seasons. All hypothesis tests were two sided. The linear-mixed model allows us to adjust for potentially confounding covariates such as gender, age, and body mass index (BMI) in the analysis. Both unadjusted and adjusted *P* values for gender, age, and BMI are reported. It should be noted that the comparisons between the seasons using the linear-mixed model were predominately based on the repeated seasonal measurements within single individuals. This is a robust method that effectively allows for the assessment of data when missing data points are present (Verbeke and Molenberghs 2000). Pearson correlation was used to assess the relationships within the cardiovascular variables between seasons. The subgroup for each correlation between two seasons (e.g., winter vs. spring) included all subjects who were studied in both seasons, and did not include the subjects who were studied in only one of the two seasons. A paired *t*-test was employed to further verify the differences in the variables between the seasons (e.g., winter vs. spring) in the subgroup of subjects for the correlation analysis. All statistical analyses were performed using SAS software (SAS Institute Inc., Cary, NC). Data are reported as means ± SD.

## Results

**Figure 1:**
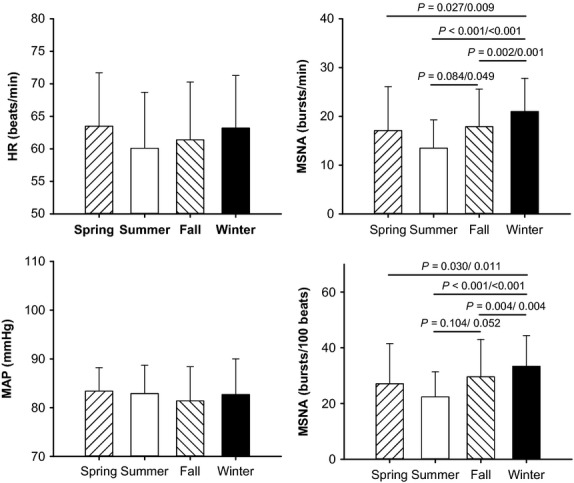
Heart rate (HR), mean arterial blood pressure (MAP), MSNA burst rate (right upper panel), and MSNA burst incidence (right lower panel) in the four seasons. Data are reported as means ± SD. Subject numbers for each season are reported in Table1. Unadjusted *P* value for the seasons: *P *=* *0.109 for HR, *P *=* *0.656 for MAP, *P *<* *0.001 for MSNA burst rate, and *P *<* *0.001 for MSNA burst incidence. After adjusting for gender, age, and BMI, *P *=* *0.117 for HR, *P *=* *0.652 for MAP, *P *<* *0.001 for MSNA burst rate, and *P *<* *0.001 for MSNA burst incidence. *P* values in the figure (e.g., *P *=* *0.027/0.009) were the unadjusted value and the adjusted value for gender, age, and BMI, respectively.

The mean age, body weight, and BMI of all subjects studied did not vary as a function of season (Table1). Figure1 shows the average MSNA burst rate and incidence, heart rate, and MAP in all subjects in the four seasons. MSNA in the winter was greater than during the other seasons. After adjusting for the gender, age, and BMI, the differences were still significant. There were no significant differences in MSNA burst rate or incidence for other seasonal comparisons. It should be noted that the mean values and the standard deviation in Table1 and Figure1 for each season were from the data of all subjects studied in the season, while the comparisons between the seasons using the linear-mixed model were predominately based on the repeated seasonal measurements within single individuals. When the data obtained from females (*N *=* *25) and males (*N *=* *35) were analyzed separately, a seasonal effect on MSNA was observed for both genders. Specifically, winter values of MSNA burst rate (male: 20.5 ± 6.9 vs. 13.8 ± 5.5, *P *<* *0.001; female: 21.8 ± 6.7 vs. 13.0 ± 6.4, *P *=* *0.014) and incidence (male: 33.9 ± 12.2 vs. 23.5 ± 8.9, *P *<* *0.001; female: 33.6 ± 9.3 vs. 20.9 ± 9.1, *P *=* *0.019) were greater than the summer values. However, statistical differences for other seasonal comparisons were lost.

**Figure 2:**
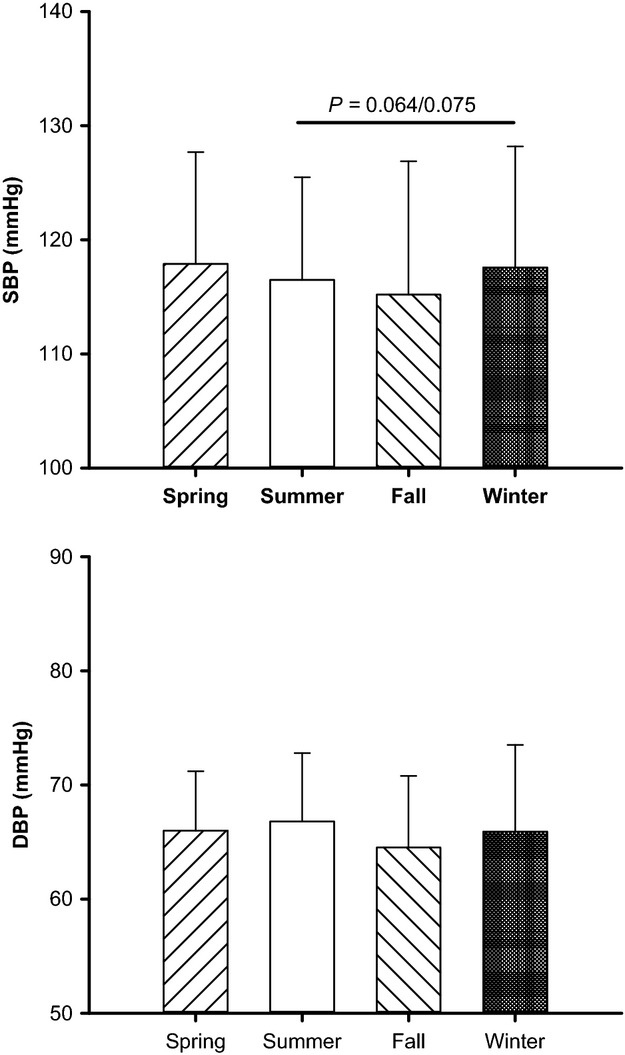
Systolic (SBP) and diastolic (DBP) blood pressure in the four seasons. Subject numbers for each season are reported in Table1. Unadjusted *P* value for the seasons: *P *=* *0.043 for SBP and *P *=* *0.286 for DBP. After adjusting for gender, age, and BMI, *P *=* *0.056 for SBP and *P *=* *0.264 for DBP.

SBP and DBP in all subjects in the four seasons are shown in Figure2. Heart rate, DBP, and MAP did not differ with season. The unadjusted analysis showed that SBP significantly varied with the season (*P *=* *0.043). After adjusting for gender, age, and BMI, SBP tended to vary with the season (*P *=* *0.056). The *P* values for the comparison of SBP between the winter and the summer from the post hoc analysis were 0.064 and 0.075 for the unadjusted and adjusted values, respectively.

**Figure 3:**
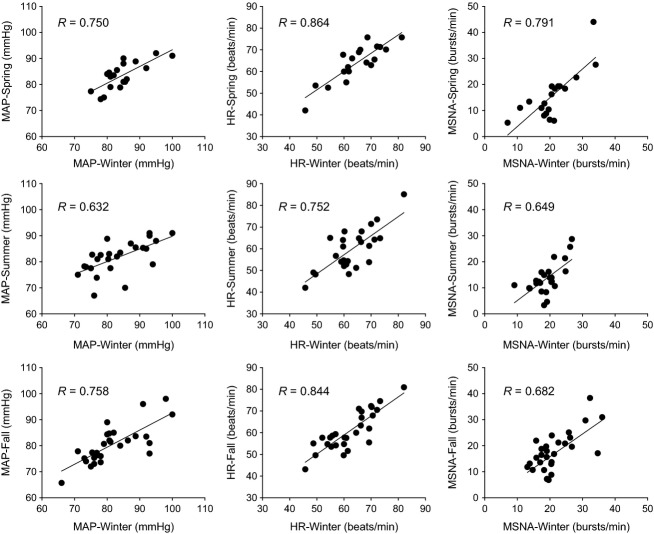
Relationships within MAP, HR, and MSNA between the winter and other three seasons. Each dot in the graphs represents the variable observed in two seasons in an individual subject. The subjects for each comparison of the seasons were the same. The subgroups of the subjects in the upper, middle, and lower panels were not the exact same, but had overlap. *N *=* *19 for winter to spring. *N *=* *25 for winter to summer. *N *=* *28 for winter to fall.

In Figure3, we examined the relationship within heart rate, MAP, and MSNA during different seasons in each of the subjects. MSNA, heart rate, and MAP correlated with each other. In each of these subgroups in Figure3, MSNA in the winter was still significantly higher than in the other seasons. The comparisons of MSNA for the subgroups of the subjects in the upper panel, the middle panel, and the lower panel in Figure3 were 20.7 ± 6.6 versus 15.7 ± 9.2 bursts/min (winter vs. spring, *P *=* *0.001, *N *=* *19, 9 M, 10 F), 19.9 ± 5.0 versus 13.8 ± 6.0 bursts/min (winter vs. summer, *P *<* *0.001, *N *=* *25, 15 M, 10 F), and 21.5 ± 6.1 versus 17.9 ± 7.3 bursts/min (winter vs. fall, *P *=* *0.002, *N *=* *28, 19 M, 9 F), respectively. The comparisons of weight for these three subgroups of subjects were 71.5 ± 11.8 versus 71.3 ± 11.8 kg (winter vs. spring, *P *=* *0.68, *N *=* *19), 76.0 ± 11.7 versus 75.8 ± 12.5 kg (winter vs. summer, *P *=* *0.84, *N *=* *25), and 78.0 ± 12.1 versus 77.6 ± 12.5 kg (winter vs. fall, *P *=* *0.84, *N *=* *28), respectively. For the subgroup of the subjects who were studied in the winter and the summer (the middle panels in Fig.3), the SBP was 120 ± 11 versus 116 ± 9 mmHg (*P *<* *0.01, *N *=* *25).

## Discussion

Our main finding in this report is that resting MSNA activity is higher in the winter than it is in the other seasons. We suggest that the seasonal changes in sympathetic activity may be a contribution to the previously observed seasonal variations in cardiovascular morbidity and mortality.

The main finding in this report was based on the analysis using a linear-mixed effects model, which is a robust method that effectively allows for the assessment of data when missing data points are present (Verbeke and Molenberghs 2000). In fact, the comparisons between the seasons using the linear-mixed model were predominately based on the repeated seasonal measurements within single individuals (see the *N* numbers in Fig.3). Although only a few subjects were studied in all of the four seasons, the main finding was predominately based on the data from more subjects.

Within a given season, large interindividual differences in MSNA (see Fig.3) were observed among the individuals. This is not surprising as interindividual difference in MSNA was due to many factors including age, gender (Ng et al. 1993), BMI (Scherrer et al. 1994), and genetics (Wallin et al. 1993). Moreover, lifestyle factors such as daily physical activities and diet, etc., may directly and/or indirectly affect MSNA. On the other hand, the data shown in Figure3 show that compared with the interindividual difference the intraindividual variability was relatively small. This is also consistent with prior observations that resting MSNA values for a given subject remains relatively stable and reproducible when measured on several occasions separated over months and even years (Fagius and Wallin 1993; Wallin and Elam 1994; Hoffman et al. 1998). Moreover, this intraindividual stability suggests, at least to some extent, that the variables measured were reproducible in multiple projects from years of data collection. Thus, we believe this intraindividual stability adds to the validity of our observations regarding the effects of season on MSNA.

We believe we can exclude a number of factors as potentially contributing to the observed effects of season on MSNA. First, the effects we have seen are not due to a systematic effect of aging, which increases basal MSNA (Fagius and Wallin 1993; Ng et al. 1993). The winter studies were not systematically done in the later years of our report, and there was no significant difference in the age between the different seasonal data sets. Second, the seasonal effects were not due to seasonal effects on body weight or BMI (Scherrer et al. 1994). Subjects’ weights in the different seasons were similar. Moreover, after adjusting the factors of gender, age, and BMI in the statistical analysis, the seasonal differences were still significant (Fig.1). Of note, the differences in the weight seen in Table1 were predominately due to the fact that the subjects were not exactly the same for the four seasons, while the comparisons between the seasons using the linear-mixed model were predominately based on the repeated seasonal measurements within single individuals. When the differences in the weight were further examined within the subgroups of the subjects in Figure3, the differences in the weight between the seasons were small.

Our results were not caused by short-term rhythmic fluctuations such as respiratory (at approximate 4-sec period) or Mayer rhythms (at approximate 10-sec period) (Furlan et al. 2000). These possibilities should be excluded by using a 6-min baseline measurement period. The effect we observed was not likely due to circadian influences as the overwhelming majority of our experiments were performed in the morning (209/237 visits). Moreover, a prior study showed that MSNA burst rate is the same in the morning as it is in the afternoon (Middlekauff and Sontz 1995).

Gender also influences the autonomic activity (Evans et al. 2001). Moreover, it has been shown that the gender influences the sympathetic responses to many different physiological interventions (e.g., chemoreflex stress, exercise) (Jarvis et al. 2011; Usselman et al. 2015). The sympathetic responses to other interventions such as the cold presor test (Jarvis et al. 2011) are not influenced by gender. Over half of the subjects included in this report were men and thus systematic menstrual cycle effects did not lead to our seasons MSNA effects. In the studies for each season, the female subjects had different menstrual cycle phases. Moreover, the observations for the potential effects of the menstrual cycle on resting MSNA in previous reports are controversial (Minson et al. 2000; Carter et al. 2009). Of note, when we separately analyzed male and female data, we still observed a seasonal effect on MSNA that the winter values were significantly greater than those in the summer in both groups. Future prospective studies controlling for phase of the menstrual cycle will be needed to examine this issue.

In general terms, it is not clear how season influences cardiovascular system. Prior reports have suggested a number of factors. These include seasonal differences in outdoor (Barnett et al. 2007; Alperovitch et al. 2009; Kent et al. 2011) and indoor (Woodhouse et al. 1993; Barnett et al. 2007; Alperovitch et al. 2009) temperatures, the hours of daylight (Modesti et al. 2013), seasonal differences in physical activity (Dannenberg et al. 1989), and seasonal variation in subjects’ emotional state (Kasper et al. 1989). For example, studies on seasonal variations in BP suggest that the main factor is a difference in temperature (Alperovitch et al. 2009; Halonen et al. 2011; Kent et al. 2011), that may affect the central sympathetic tone (Brennan et al. 1982). We speculate that these factors (e.g., temperature, daylight, etc.) contribute directly and/or indirectly (e.g., via daily physical activity) to the observed seasonal variation in resting MSNA, since the outdoor temperature and hours of daylight distinctly vary with season in the area of the data collection in this report. One prior report suggests that people living in southern latitudes show a considerable seasonal variation in BP (Barnett et al. 2007). Thus, we speculate that our observations are potentially relevant to the population in the geographic zones, where the similar seasonal variations in temperature and daylight are presented.

The seasonal variations in resting MSNA that we note are consistent with previous observations, which suggest that various indices of sympathetic activity are higher in the winter than the summer. A prior preliminary report with eight subjects by Niimi et al. (Niimi et al. 1999) suggested that MSNA might be higher in the winter than in the summer. No data for the other seasons were discussed in that report. Plasma norepinephrine tended to be higher in the winter than in the summer (Izzo et al. 1990; Radke and Izzo 2010). Although catecholamines provide more global data than do measurements of MSNA, MSNA provides direct sympathetic activity and thus provide unique data. Nevertheless, there are clear relationships between MSNA and blood catecholamines. For example, Victor and colleagues (Victor et al. 1987) showed strong correlation between the MSNA response and BP response as well as a strong correlation between MSNA and plasma norepinephrine responses to the cold pressor test. Moreover, an inverse correlation has been demonstrated between MSNA and cardiac output, and MSNA correlates with indices of total peripheral resistance (Wallin 2007). It has been shown that total peripheral resistance was higher while cardiac output was lower in the winter (Izzo et al. 1990). Kristal-Boneh et al. have shown that the heart rate variability was lower in the winter than in the summer (Kristal-Boneh et al. 2000).

The seasonal differences in MSNA we noted, especially for the comparisons of the winter versus the spring and the winter versus the fall, were modest. These data suggest that MSNA varies with season in a continuous gradual fashion. Moreover, it should be emphasized that the MSNA values (i.e., per minutes) we observed were reflective of resting or basal conditions. We speculate that even small changes in basal sympathetic outflow may have an impact on the cardiovascular system itself and the cardiovascular risks when the conditions last for months.

Prior reports have shown that BP tends to be higher in the winter than in the summer (Brennan et al. 1982; Woodhouse et al. 1993; Barnett et al. 2007; Alperovitch et al. 2009; Kent et al. 2011; Modesti et al. 2013). In the present report, although we did not find significant differences in DBP or MAP between the seasons, SBP tended to be higher in the winter than in the summer. In fact, for the subgroup of the subjects who were studied in the winter and the summer (the middle panels in Fig.3), the SBP in the winter was significantly higher than in the summer. Our observations are consistent with the findings from the prior large epidemiologic reports (Brennan et al. 1982; Alperovitch et al. 2009; Kent et al. 2011; Modesti et al. 2013). Interestingly, these prior epidemiologic reports suggest that the effects of season on BP seem to increase as a function of the age of the subject studied (Brennan et al. 1982; Woodhouse et al. 1993; Alperovitch et al. 2009). Moreover, the BP response to an acute cold stress is greater with age (Hess et al. 2009). The mean age of our subjects was 28. We suspect that if we had studied an older cohort of individuals we would have seen a larger effect of seasons on BP.

### Perspective

Higher winter event rates have been observed for cardiovascular diseases such as myocardial infarction (Dunnigan et al. 1970; Spencer et al. 1998; Kloner et al. 1999; Sheth et al. 1999), stroke (Sheth et al. 1999), subarachnoid hemorrhage (Nyquist et al. 2001), abdominal aortic aneurysmal rupture (Ballaro et al. 1998), and aortic dissection (Mehta et al. 2002). Moreover, seasonal variations in mortality and disease incidence have been observed for coronary heart disease (Kloner et al. 1999) and heart failure (Boulay et al. 1999; Martinez-Selles et al. 2002; Stewart et al. 2002; Diaz et al. 2007). Many of these prior reports speculated that seasonal variations in sympathetic tone contributed to these seasonal variations in the cardiovascular diseases. Our report provides direct evidence that in normal subjects basal sympathetic tone is higher in the winter than during other seasons. Further studies are needed to determine whether the seasonal variations in sympathetic tone are seen in patients with specific cardiovascular diseases. We should note that our findings do not provide insight into the mechanisms responsible for the high incidence of cardiac events seen with extremes of high temperature during heat waves (Semenza et al. 1996).

### Study limitations

We acknowledge that our study has some limitations. The records included in this article were part of other studies and thus the data in this report were examined in a retrospective fashion. This approach does not afford us the opportunity to make specific statements about the differences in lifestyle or individual environmental exposure to different climatic factors that may have contributed to our findings. The relationships between meteorological variables, life style factors, and the seasonal variation in MSNA can be examined in further studies. Nevertheless, our observations provide a potential physiologic mechanism for seasonal variations in cardiovascular risks that have been observed.

In summary, our finding that basal sympathetic nerve activity has a seasonal variation with a winter peak supports the concept that higher winter sympathetic tone may contribute to the seasonal variations in cardiovascular events. Further investigations focusing on the seasonal variation in the sympathetic responses to stressor (e.g., exercise) are warranted.
